# A 60-GHz Wideband High-Efficiency Circularly Polarized Dual-Coil Antenna Array

**DOI:** 10.3390/s25072211

**Published:** 2025-03-31

**Authors:** Jun Xiao, Qi Gan, Zihang Ye, Chong-Zhi Han, Tongyu Ding, Qiubo Ye

**Affiliations:** 1 School of Ocean Information Engineering, Jimei University, Xiamen 361021, China; xiaojun@jmu.edu.cn (J.X.); 202412854001@jmu.edu.cn (Q.G.); 202021114019@jmu.edu.cn (Z.Y.); 202061000139@jmu.edu.cn (C.-Z.H.); 2 College of Information Science and Engineering, Hohai University, Changzhou 213200, China

**Keywords:** high efficiency, circularly polarized (CP), self-sequential rotation technique, antenna array, 60-GHz

## Abstract

A wideband high-efficiency circularly polarized (CP) dual-coil antenna array is presented for 60-GHz applications in this letter. The proposed CP dual-coil antenna is composed of a resonant substrate-integrated cavity (SIC) and a pair of centrally symmetrical coils, which are fed differentially by a substrate-integrated waveguide (SIW) coupling slot. A novel sequential rotation feeding technique is introduced to enhance the axial ratio (AR) and impedance bandwidths of both the 2 × 2 subarray and the 4 × 4 array. The proposed feeding network significantly improves radiation efficiency. The measured results of the fabricated prototype indicate that the proposed array achieves an impedance bandwidth of 20.8% (54.6–67.3 GHz) for |S_11_| ≤ −10 dB, a 3-dB AR bandwidth of 21.5% (54–67 GHz), a high radiation efficiency of 96.6%, and a peak gain reaching 19.3 dBic at 58 GHz. The proposed circularly polarized (CP) antenna element and array design stand out as strong contenders for 60-GHz wireless applications.

## 1. Introduction

The unlicensed spectrum in the 60 GHz band (from 57 to 71 GHz) has garnered in-creasing research interest. This is primarily because it offers a considerable amount of available working spectrum compared to lower-frequency bands. In addition to the aforementioned benefits, 60-GHz wireless communication provides several other advantages, such as high-speed transmission, low latency, large capacity, and enhanced security [1]. However, despite these numerous advantages, there are also challenges associated with 60-GHz wireless communication. The 60-GHz signal experiences significant absorption in the atmosphere, primarily due to the higher absorption rates of water vapor and oxygen molecules for electromagnetic waves at this frequency [2]. Therefore, one approach to address this challenge is to design antennas with high gain that can be implemented in arrays.

Circularly polarized (CP) antennas play a pivotal role in facilitating 60-GHz wireless communication. Due to the susceptibility of signals in the 60-GHz band to obstacles and interference from multipath effects, the use of linearly polarized antennas can result in signal attenuation and phase distortion. However, by utilizing CP antennas, it becomes possible to achieve both horizontal and vertical polarization during reception and trans-mission. This characteristic greatly enhances signal robustness and mitigates the impact of multipath interference [3,4]. Driven by the appeal of these exceptional attributes, exten-sive investigations have been conducted on a diverse range of CP antenna elements and arrays specifically designed for 60-GHz applications [5,6,7,8,9,10,11,12,13,14,15,16], including strip shaped patched [5], magnetoelectric dipole (ME-dipole) antennas [6], S-dipoles [7], helixes [8], spirals antennas [9], complementary antennas [10,11], slits loaded circular patched [12], L-probe fed antennas [13], aperture antennas [14], and lens antennas [15,16]. In tackling the challenge of the limited 3-dB AR bandwidth in CP antennas, a sequentially rotated feeding technique has been proposed as a means to enhance the antenna’s bandwidth [17,18,19,20,21,22,23,24,25,26,27]. This technique offers a viable solution to expand the operational bandwidth of the antenna, effectively overcoming the limitations associated with a narrow AR bandwidth.

In this letter, a 60-GHz broadband CP dual-coil antenna element and a 4 × 4 array are proposed based on sequentially rotated feeding technique. The CP radiation is realized by exciting the designed two coil-shaped patched etched on the substrate. Introducing a 2 × 2 subarray with a sequential rotation feeding network is proposed to enhance both impedance and the 3-dB axial ratio (AR) bandwidth. Finally, a 2 × 2 subarray is employed to construct a sequential, rotationally fed 4 × 4 CP array with a wide impedance bandwidth of 20.8% (54.6–67.3 GHz), a 3-dB AR bandwidth of 21.5% (54–67 GHz), a high radiation efficiency of 96.6%, and a peak gain of 19.3 dBic at 58 GHz. The antenna array is fabricated using Rogers 5880 substrate. The measurement results demonstrated that the proposed antenna array attains wideband, high-efficiency, and stable radiation patterns.

## 2. Antenna Design

### 2.1. Geometry of the Element

**Figure 1:**
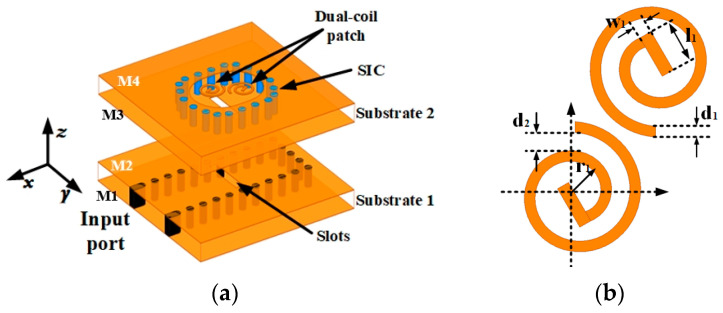

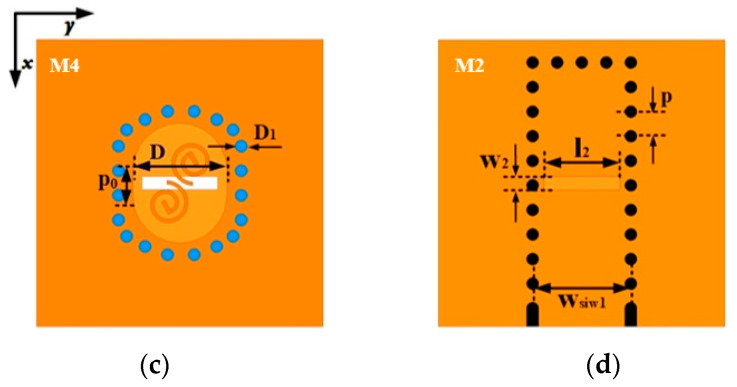
Geometry of proposed element. (**a**) Exploded view. (**b**) Top view of dual-coil. (**c**) Substrate 2. (**d**) Top view of Substrate 1. (w_1_ = 0.1, w_2_ = 0.33, w_siw1_ = 2.4, l_1_ = 0.35, l_2_ = 1.86, d_1_ = 0.085, d_2_ = 0.145, r_1_ = 0.16, D = 2.3, D_1_ = 0.3, P_0_ = 0.64, and P = 0.6, all in millimeters.).

**Figure 2:**
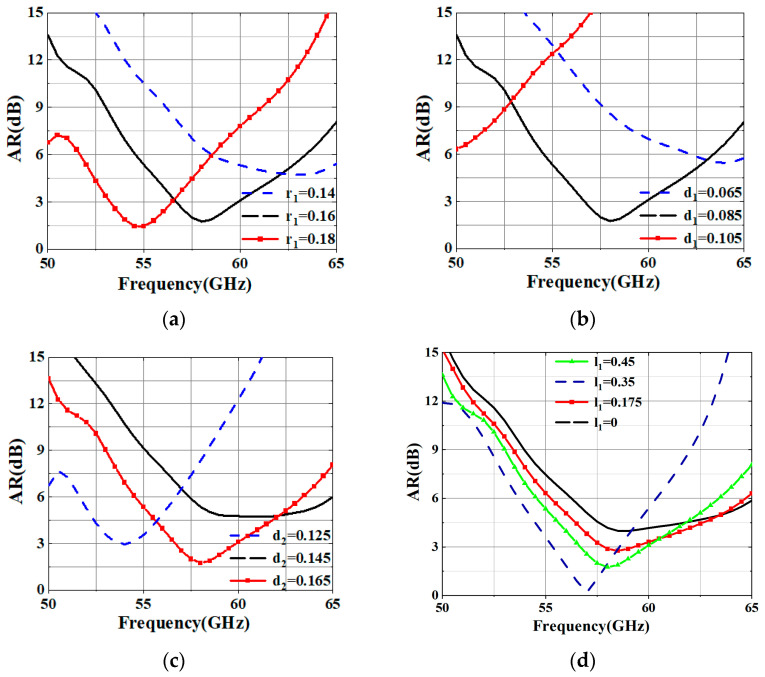
Simulated AR performances of the dual-coil with different radii, widths, and gaps: (**a**) effects of *r*_1_ on AR; (**b**) effects of *d*_1_ on AR; (**c**) effects of *d*_2_ on AR; and (**d**) effects of *l*_1_ on AR.

The geometrical structure of the designed SIW-fed CP dual-coil antenna element is illustrated in Figure 1a. It is composed of four-layer metallic laminates and two-layer dielectric substrates: M1–M4 (PEC), Substrate 1, and Substrate 2 (Rogers 5880, a relative dielectric constant of 2.2, and a tangent loss of 0.0009). The substrate thicknesses are all 0.787 mm. As illustrated in Figure 1c, the radiation part incorporates an elliptical SIW cavity, with two coil-shaped patches on the top of Substrate 2 to generate CP radiation. The introduction of straight sections in the dual-coil design optimizes the 3-dB AR bandwidth. Based on this, the precise adjustment of the radius, width, and gap of the dual-coil allows for control over the amplitudes of the two orthogonal currents, thereby further improving the circular polarization performance. The detailed structure and dimensions of the coil are shown in Figure 1b. The proposed radiator is fed through a horizontal coupling slot etched on the wide wall of the SIW in Substrate 1, as depicted in Figure 1d. We used the CST MICROWAVE STUDIO to design and simulate the proposed antenna. The AR performance is illustrated in Figure 2 according to dual-coil size variation. The detailed optimized parameters are provided in the caption of Figure 1.

### 2.2. Element-Working Principle

**Figure 3:**
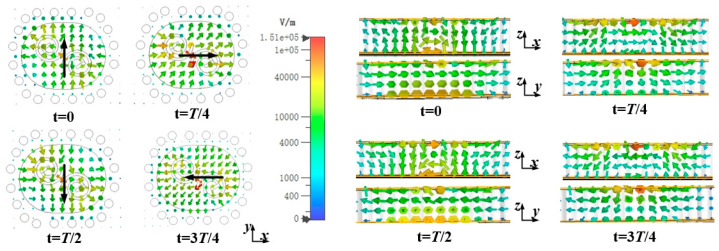
E-field distributions on the proposed antenna element at 60 GHz over a period of time.

**Figure 4:**
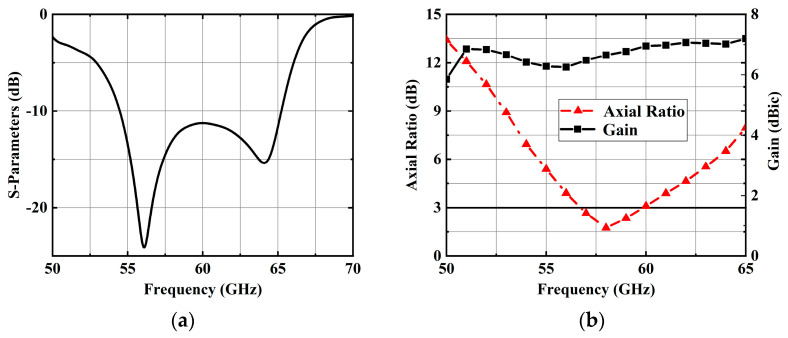
Simulated results of the proposed CP dual-coil antenna elements. (**a**) Simulated |S_11_|. (**b**) Simulated ARs and gains.

Figure 3 depicts the electric field distribution at four distinct states during the operation of the proposed antenna element, elucidating its operational mechanism. These states are specifically captured at time instants t = 0, *T*/4, *T*/2, and 3*T*/4, where *T* = 1/(60 × 10^9^) seconds is the period corresponding to a frequency of 60 GHz. The energy primarily radiates through the aperture formed by the Substrate Integrated Cavity (SIC) and the dual-coil patches from the coupling slot. Arranging a coil pair appropriately generates electric fields in both *x*- and *y*-polarizations within the radiating aperture. For achieving CP radiation, it is essential to ensure equal amplitude and a quadrature phase difference between the *x*- and *y*-components of the electric field. When observed along the direction of electromagnetic wave propagation, since the *x*-component leads the *y*-component by 90 degrees and both have equal amplitudes, the electric field vector exhibits a clockwise rotation over time. By appropriately choosing the parameters, the desired CP radiation can be effectively produced. The black arrow on the antenna’s surface indicates the direction of the electric field vector, displaying a clockwise rotation over time. This rotation results in the generation of a left-hand circularly polarized wave. Figure 4 depicts the experimental results of the antenna element.

### 2.3. Antenna Element Performance

The suggested CP antenna element exhibits a simulated impedance bandwidth (|S_11_| ≤ −10 dB) of 17.9% (54.5–65.2 GHz) and a simulated 3-dB AR bandwidth of 5.5% (56.7–59.9 GHz). Moreover, the antenna element attains a peak gain of 7.20 dBic at 65 GHz. Notably, the antenna element demonstrates stable gain. These characteristics emphasize the suitability of the suggested antenna element for constructing high-gain and wideband antenna arrays.

## 3. Antenna Array Design

### 3.1. 2 × 2 Subarray

**Figure 5:**
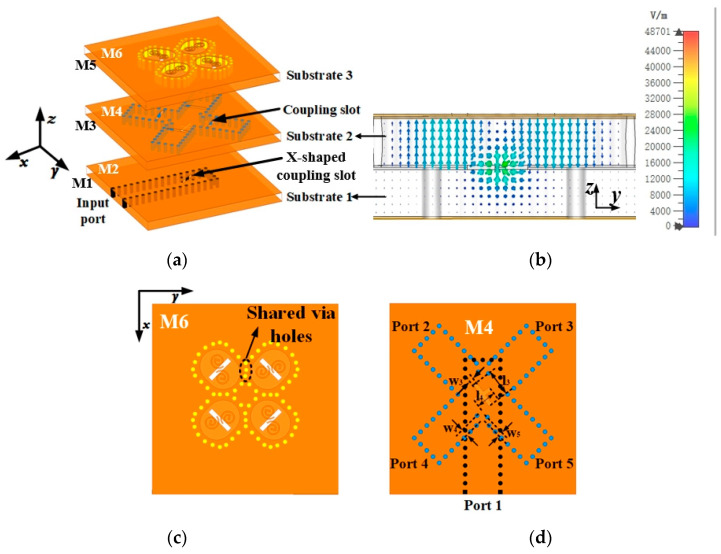
Geometry of the proposed subarray. (**a**) Exploded view. (**b**) E-field intensity distribution in the SIW structure of Substrate 1 and 2 (**c**) top view. (**d**) Top view of feeding network.

**Figure 6:**
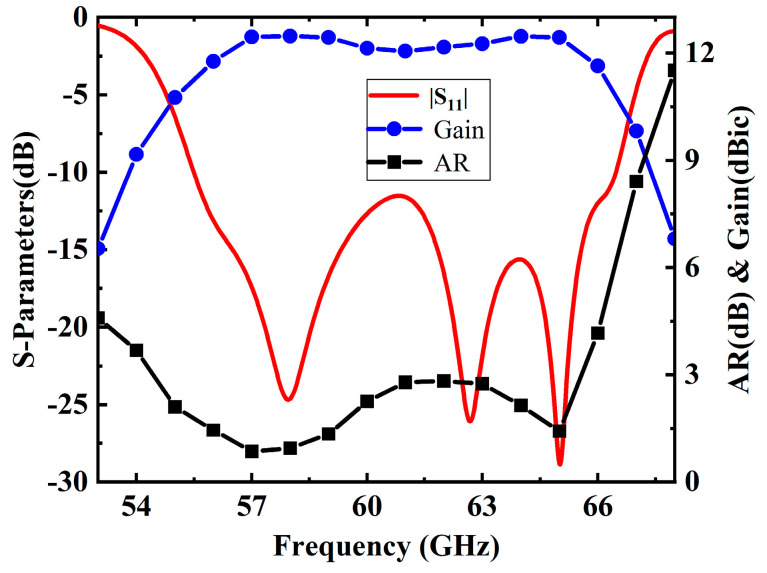
Simulated |S_11_|, gain, and AR of the 2 × 2 CP dual-coil antenna subarray.

**Figure 7:**
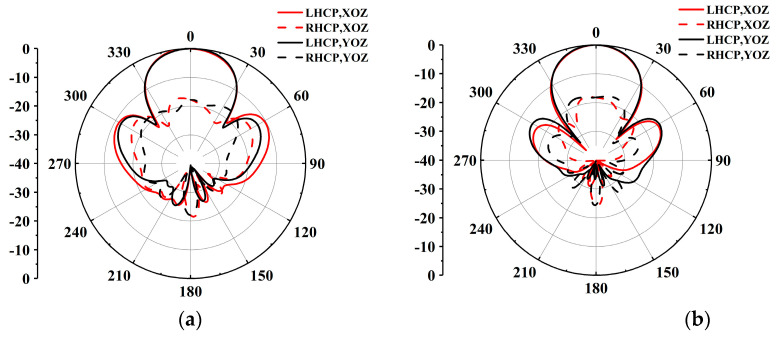
Radiation patterns of 2 × 2 CP dual-coil antenna subarray at (**a**) 60 GHz and (**b**) 64 GHz.

Utilizing the proposed CP antenna element, a 2 × 2 CP antenna subarray is meticulously crafted, as illustrated in Figure 5. The subarray incorporates a wideband one-to-four power divider with dual layers of 0.787 mm Rogers 5880 substrate. Energy is coupled from the Substrate 1 to the Substrate 2 through an X-shaped coupling slot. The energy transfer path from Substrate 1 to Substrate 2 is clearly shown in Figure 5b. To achieve wide impedance and 3-dB AR bandwidths, a sequential rotation feeding network is introduced in Substrate 2. However, the SIW structure is prone to resonances, which can cause fluctuations in the frequency response and potentially degrade performance. The four port outputs demonstrate roughly equal magnitude, and a 90° phase difference can be achieved by adjusting the position of the impedance matching post and optimizing the size of the X-shaped coupling slot on the top of Substrate 1. The simulated results for the 2 × 2 CP subarray are shown in Figure 6, with radiation patterns depicted in Figure 7.

The simulated impedance bandwidth (|S_11_| ≤ −10 dB) of the subarray is 16.5% (55.5–66.5 GHz), and the simulated 3-dB AR bandwidth is 18.7% (54.4–65.6 GHz). The subarray attains a simulated peak gain of 12.5 dBic at 57 GHz.

### 3.2. 4 × 4 Array

**Figure 8:**
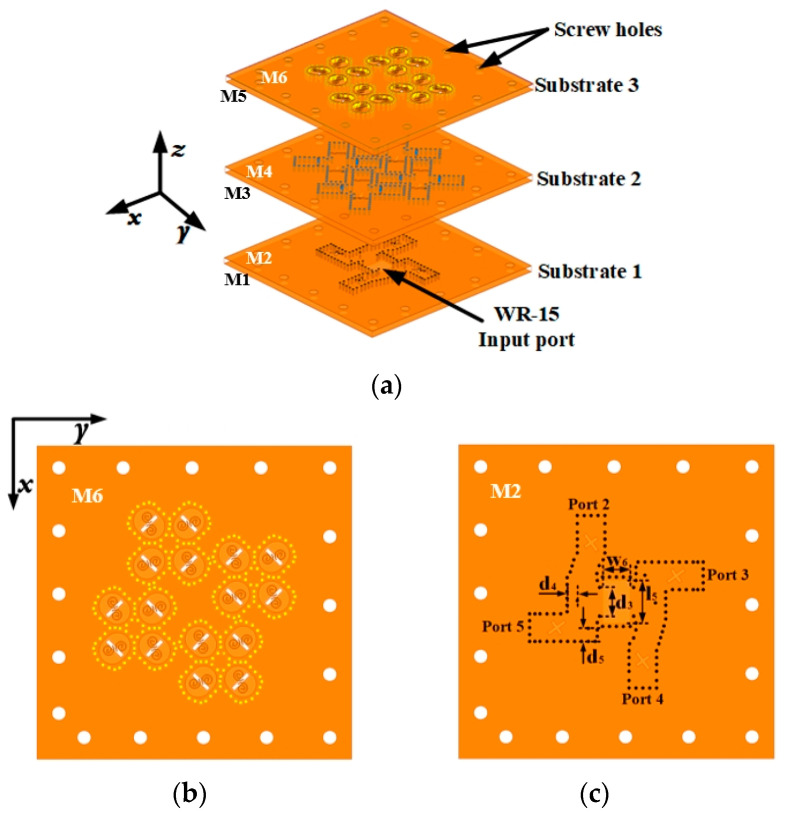
Geometry of the proposed array. (**a**) Exploded view. (**b**) Top view. (**c**) Top view of feeding network.

The proposed 2 × 2 CP subarray serves as the foundation for the design, fabrication, and measurement of the 4 × 4 CP array. The geometry of the proposed the 4 × 4 CP array is shown in Figure 8. The detailed dimensions of the 2 × 2 and 4 × 4 CP arrays are provided in Table 1. The sequential rotation feeding network, situated on Substrate 1, is utilized to stimulate four 2 × 2 subarrays, as depicted in Figure 8. The array is differentially fed by a standard WR-15 rectangular waveguide input, which operates in the TE_10_ mode for signal transmission and provides a direct connection to a differential power divider integrated into the sequential rotation feeding network. The adjacent SIC elements of the 2 × 2 subarray share a row of vias, which serves the purpose of reducing the element spacing and simplifying the array processing while maintaining good isolation performance. All three substrate board layers employ Rogers RT5880 substrates, each with a thickness of 0.787 mm.

## 4. Measurement and Comparisons

**Figure 9:**
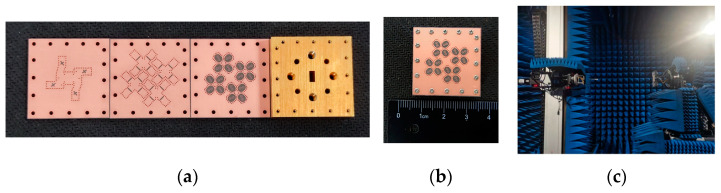
Prototype of the proposed antenna array. (**a**) Fabricated disassembled prototype. (**b**) Top view of the assembled antenna array. (**c**) Far-field radiation test setup for proposed CP array.

Figure 9 depicts the fabrication of a 4 × 4 CP antenna array using PCB technology. The antenna assembly is affixed using screws strategically placed around the antenna, with the screw holes serving purely for assembly and having a negligible impact on the antenna’s performance. The prototype has a compact size, measuring 27.3 mm × 27.4 mm.

### 4.1. Experimental Results

**Figure 10:**
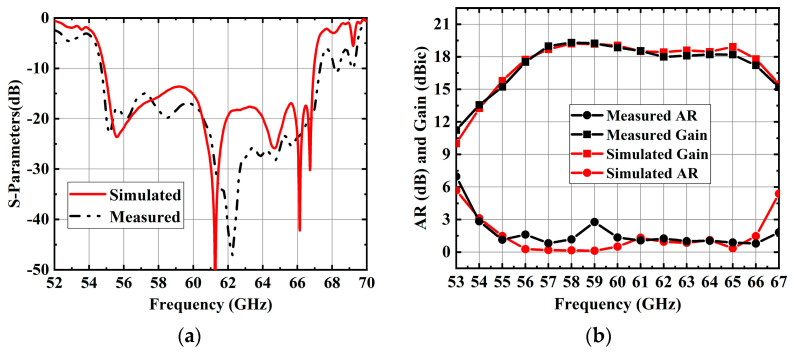
Simulated and measurement results of the proposed 4 × 4 antenna array. (**a**) |S_11_|. (**b**) AR and gain.

Figure 10 depicts the experimental results of the 4 × 4 antenna array. The impedance bandwidth of the 4 × 4 antenna array was simulated to be 19.9% (54.9–67.0 GHz) and measured to be 20.8% (54.6–67.3 GHz). Similarly, the simulated 3-dB AR bandwidth was 20.4% (54.1–66.4 GHz), while the measurement 3-dB AR bandwidth was slightly wider at 21.5% (54–67 GHz). During measurements, the antenna exhibited a peak gain of 19.3 dBic at 58 GHz. The radiation efficiency can be measured by comparing the simulated directivity with the measured gain. The maximum radiation efficiency of the 4 × 4 array at 58 GHz was 96.6%.

**Figure 11:**
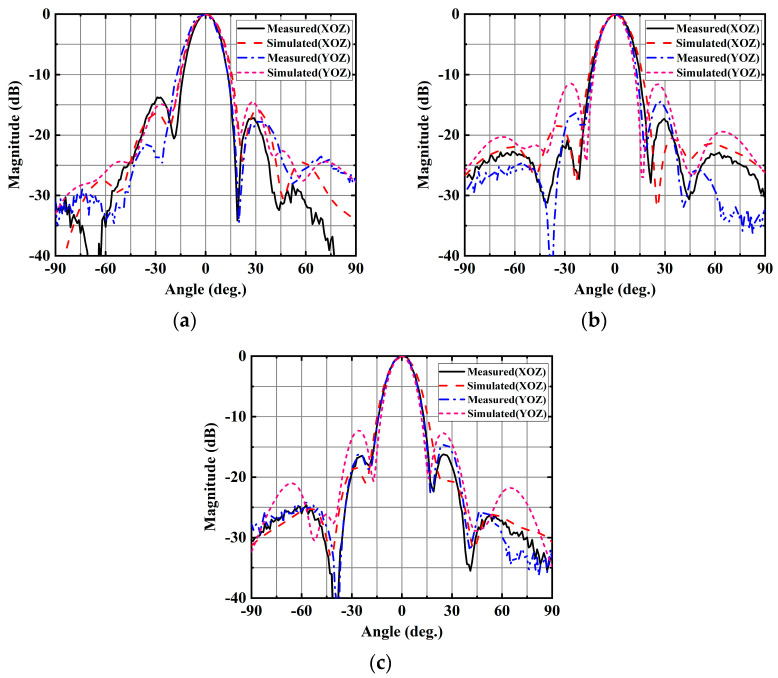
Simulated and measurement normalized radiation pattern of the 4 × 4 CP antenna array. (**a**) 56 GHz. (**b**) 60 GHz. (**c**) 64 GHz.

Furthermore, Figure 11 portrays the simulated and measurement normalized radiation patterns in the xoz and yoz planes at frequencies of 56, 60, and 64 GHz. Notably, the radiation patterns of both the simulated and measurement results remain stable and consistent throughout the entire operating band.

### 4.2. Comparisons and Discussion

Table 2 offers a detailed overview of the proposed design’s performance metrics and key parameters, alongside data from various previously published CP antenna arrays. As depicted in Table 2, the proposed work achieves the highest maximum radiation efficiency of 96.6% among the recently reported CP arrays. While the 4 × 4 CP array presented in [28] achieves a higher peak gain compared to the proposed array, the proposed CP array based on the sequentially rotated feeding network surpasses it with a 9.5% higher maximum radiation efficiency, reaching an impressive value of 96.6%. Additionally, the proposed array offers a wider 3-dB AR bandwidth and impedance bandwidth, further enhancing its performance capabilities. While the 4 × 4 array presented in [29] offers a slightly wider bandwidth compared to the proposed CP array, it is important to note that the maximum radiation efficiency of the proposed array surpasses it by a significant margin of approximately 38.7%. The antenna proposed in this communication stands out when compared to the 60-GHz antenna array in [6,8,9], as it not only exhibits a wide bandwidth but also demonstrates exceptional maximum radiation efficiency.

Considering factors such as impedance bandwidth, 3-dB AR bandwidth, radiation efficiency, and the gain of the antenna, a thorough comparison of the results highlights the proposed design as a highly promising choice for a high-performance CP antenna array tailored for 60-GHz wireless applications. When scaling up to an 8 × 8 or larger array configuration, the complexity of the feed network design and the coupling effects among components will increase significantly. This may give rise to challenges in impedance matching. Nevertheless, the advantage is that it enables the achievement of higher gain.

## 5. Conclusions

In this letter, we present an innovative design of a dual-coil patch antenna array for 60-GHz applications. The proposed novel geometrical structure validates that the combination of SIC and dual-coil patches enables the antenna array to achieve wideband and high radiation efficiency. The 4 × 4 antenna array, utilizing an SIW sequentially rotated feeding network, demonstrates broadband and high-efficiency performance. The CP array design exhibits considerable promise for applications in 60-GHz wireless communications, delivering wideband performance with a simplified structure.

## Figures and Tables

**Table 1:** Parameters of the antenna array (unit: mm).

Para	Value	Para	Value	Para	Value
w_3_	0.3	w_4_	0.35	w_5_	0.36
w_6_	2.38	l_3_	1.8	l_4_	1.34
l_5_	3.759	d_3_	2.5	d_4_	0.89
d_5_	1.14				

**Table 2:** Comparison with the reported antenna arrays.

Ref.	Element Type	No. of Element	Feeding Network	*f*_0_ (GHz)	Imp. BW	3-dB AR BW, %	Peak Gain (*dBic)	Max. Radiation Efficiency (%)	Total Size (λ_0_ × λ_0_ × λ_0_)
[5]	Cavity-back slot-dipole	8 × 8	SIW	29	21.9	22.07	26.1	72.54	6.68 × 6.68 × 0.27
[6]	ME-dipole	8 × 8	SIW	60	18.2	16.5	26.1	72.20	6.22 × 6.92 × 0.50
[7]	Slot-coupled S-dipole	8 × 8	SIW	30	27.6	32.7	25.2	75.70	5.90 × 5.90 × 0.47
[8]	Helical antenna	4 × 4	Microstrip line	60	22	20	15.2	54.90	2.00 × 2.40 × 0.40
[9]	Slot-coupled spirals	4 × 4	SIW	60	16.6	>18.8	20	63.86	6.0 × 6.0 × 0.48
[17]	Dipole with hexagonal parasitic patched	4 × 4	SIW	30.5	27.7	28.5	17.85	*n.a.	*n.a.
[28]	Spiral patch	4 × 4	SIW	60	14.1	21.1	19.5	>77	4.8 × 4.8 × 0.49
[29]	Cavity-back stacked patches	4 × 4	CPW	29	29.6	25.4	20.32	>57.9	10.2 × 8.7 × 0.098
[30]	Dual elliptical patches	4 × 4	SIW	26	31.5	24	21	90	6.07 × 6.07 × 0.61
**This work**	**Dual-coil patches**	**4 × 4**	**SIW**	**60**	**20.8**	**21.5**	**19.3**	**96.6**	**5.46 × 5.48 × 0.51**

* dBic = 10 log (antenna radiation intensity [W/Ω]/circularly polarized isotropic source [W/Ω]. *n.a.: not available in the referenced work.

## Data Availability

No new data were created during this study. The data underpinning the research findings were acquired from [specify the original source, e.g., a previous research project, a private organization’s database]. Owing to strict privacy regulations, such as those mandated by [mention the relevant privacy laws or ethical frameworks, e.g., GDPR in the European Union], and the crucial need to safeguard the identities of the individuals involved, these data cannot be shared publicly. Each data point is intricately linked to personal information, and any attempt at public disclosure could potentially lead to the identification and subsequent harm of these individuals. To ensure full compliance with privacy–related obligations and uphold the highest ethical standards in research, we have refrained from making the data accessible in a public domain.

## Abbreviations

The following abbreviations are used in this manuscript:

CP	Circularly polarized
SIC	Substrate-integrated cavity
SIW	Substrate-integrated waveguide
AR	Axial ratio
